# Investigating insulin’s role in regulating local aromatase in growth plate development

**DOI:** 10.1371/journal.pone.0337215

**Published:** 2025-12-02

**Authors:** Sicui Hu, MinMin Xue, Yao Dong, Cheng Li, Lingyan Qiao, Rongxiu Zheng

**Affiliations:** 1 Department of Pediatrics, General Hospital of Tianjin Medical University, Tianjin, China; 2 Department of Paediatric Endocrinology and Metabolism, Qingdao Women and Children’s Hospital, Qingdao, China; 3 Health Medicine Department, 983 Hospital of Joint Logistics Support Force, PLA, Tianjin, China; 4 Department of Pharmacy, Qingdao Shinan District People’s Hospital, Qingdao, China; Bowen University, NIGERIA

## Abstract

**Introduction:**

Obesity-associated hyperinsulinemia is hypothesized to disrupt linear growth trajectories, though the mechanisms remain unclear. This study investigates these mechanisms using a high-fat diet-induced rat model.

**Methods:**

Twelve healthy 1-week-old Sprague-Dawley rats (60–80 g) were randomized into two groups: a control group fed a standard diet and an obese group fed a high-fat diet. After 6 weeks of dietary intervention, weekly body weight and naso-anal length were recorded. At the end of the study, all rats were euthanized, and blood samples were collected for further analysis. Serum biochemical parameters, including insulin levels, were measured by ELISA. Tibiae and humeri were harvested for bone length measurement. Growth plates were isolated for histological analysis, immunohistochemistry, radioimmunoassay, PCR, and Western blotting.

**Results:**

Higher serum insulin levels were observed in the obese group than in the control group (36.46 ± 1.69 mU/L, vs 22.96 ± 1.99 mU/L, p < 0.01). The obese group showed higher growth plate length (426.63 ± 6.28 μm, vs 331.13 ± 28.93 μm, p < 0.01), especially in proliferative and hypertrophic areas than the control group. Immunohistochemistry analysis revealed positive staining for brownish-yellow granules in these regions. Higher aromatase levels and insulin receptor (IR) expression were observed in the growth plates, primarily in the hypertrophic region. Immunohistochemical staining indicated that aromatase expression was primarily localized to the hypertrophic zone in obese rats.Moreover, the aromatase activity in the obese group was significantly higher than that in the control group (47.29 ± 0.87 U, vs 41.12 ± 1.50 U,p ＜ 0.01). The relative mRNA expression of CYP19A1 and insulin receptors (IR) in the growth plates of obese rats was significantly higher than that in the control group (0.0039 ± 0.0026-, vs 0.0001 ± 0.00025, p < 0.01 and 0.15- ± 0.07, vs 0.03 ± 0.02, p < 0.01). Western blot analysis revealed higher expression of CYP19A1 and IR in the growth plates of obese rats (1.81 ± 0.12-, vs 1.02 ± 0.04-, p < 0.01 and 2.05 ± 0.34, vs 1.04 ± 0.13-, p < 0.01).

**Conclusion:**

In this study, we found a significant increase in the expression of insulin receptors, indicating that insulin signaling regulates aromatase expression, thereby affecting epiphyseal growth plate development. However, the molecular mechanisms by which insulin receptors regulate aromatase expression remain unclear.

## Introduction

Childhood obesity has emerged as a global issue, linked to lifetime adverse health consequences, with its prevalence increasing sharply in recent years [1]. Obese children often exhibit taller stature due to accelerated BA maturation, which may ultimately limit their optimal adult height^2^. Mounting evidence has reported that childhood obesity is often associated with an improper increase in growth rate and BA, leading to premature maturation of epiphyseal growth plates [2–4]. The influence of obesity on advanced BA is associated with factors such as BMI or total body fat mass, increased levels of estrogen, insulin-like growth factor-1 (IGF-1), etc [3].

Skeletal development is often considered an important factor during childhood and adolescence, which is influenced by complex hormonal interactions including estrogen, androgen, thyroidal and growth hormones. Among them, estrogen is reported to play a crucial role in skeletal homeostasis, regulating the senescence of the growth plate, via estrogen receptor-α (ER-α) [3,5]. Mounting evidence suggests that sexual steroids (estrogen or androgens) which have a dual effect on growth, stimulate rapid growth and are also responsible for the fusion of growth plates [5–7]. Notably, a study reported that estrogen can lead to irreversible depletion of progenitor cells in the resting zone, thereby accelerating epiphyseal fusion [8,9].Aromatase (cytochrome P450 19A1), a cytochrome P450 enzyme facilitating the conversion of androgens to estrogens and is expressed in various tissues including the ovary, testis, adipose tissue and others [10]. Its activity is primarily influenced by insulin. A study by Gibb et al reported that inhibiting aromatase reduced insulin sensitivity in humans [11,12], implying the relationship between insulin and aromatase. The expression of aromatase in epiphyseal chondrocytes was found to be upregulated during growth plate maturation in rats, suggesting its significant role in this process.

Prepubertal obese children were reported to have higher serum insulin levels, which were strongly correlated with their increased height [13]. Although the above studies describing an association between insulin resistance and height growth in humans, they did not demonstrate the interlink among insulin resistance, hyperinsulinemia and accelerated growth. Children in their prepubertal and pubertal stages have higher sexual steroids (estrogen or androgens), which have a dual effect on growth:stimulating rapid growth and promoting growth plate fusion [9,14]. For instance, a study reported that estrogen causes irreversible depletion of progenitor cells in the resting zone, thus accelerating growth plate fusion [7]. To explore the mechanistic relationship between insulin, aromatase and obesity-linked accelerated growth, we aim to investigate whether the binding of insulin to its receptor, while promoting linear bone growth, leads to advanced bone age through upregulation of aromatase expression.

## Materials and methods

### Animals

For the in vivo study, 1-week-old, healthy Spargue-Dawley (SD) rats (n = 12), weighing 60-80g were procured from the Animal center laboratory of China Three Gorges university, and were housed in standard laboratory conditions (22 ± 2℃, light: dark cycle of 12:12 h; Relative humidity: 30–70%) with standard pellet diet. After one week of adaptive feeding, all the rats were marked (via ear-tagging) and were randomized into an obese group (n = 6) and a control group (n = 6).The obese group received a high-fat diet (HFD) whereas the control group received a standard rodent diet.The control group received a standard rodent diet (4% fat, 54.6% carbohydrate, 18.2% protein; 2.9 kcal/g; Proton Bio-Technology Co., Ltd., China), while the high-fat diet (HFD) group was fed a lard-based purified diet (60% fat, 20% carbohydrate, 20% protein;5.25kcal/g; Proton Bio-Technology Co., Ltd., China).Whole-body length and weight were measured weekly, during the 6-week induction period and after its completion. Following the 6-week induction period, all the animals were euthanized using the AVMA-approved carbon dioxide asphyxiation procedure. Blood was collected to assess the serum insulin levels using ELISA, whereas bones such as the tibia and humerus were harvested to measure bone length. Growth plates were isolated and stained with hematoxylin and eosin (Wuhan Baiqiandu Biotechnology Co., Ltd., China) for analysis. All the samples were cryopreserved for PCR analysis, immunohistochemistry, radioimmunoassay and western blot analysis.

### Ethics statement

The study was approved by the Ethics Committee of the General Hospital of Tianjin Medical University(Approval no.IRB2025-DW-30).

### Immunohistopathological analysis

Histological evaluations were performed using an upright light microscope (Nikon, Japan) on pathological sections prepared with a microtome (Leica Instruments Co., Ltd., Shanghai, China).. At the end of the study, tibia and humerus were isolated and dissected to harvest growth plates. Three 5–7 mm-thick longitudinal sections were obtained from rat tibiae, which were deparaffinized in xylene and rehydrated in graded ethanol. Sections were incubated in citric acid antigen retrieval the buffer (pH 6) and heated for 5 min to prevent excessive evaporation of buffer. Slides were washed three times with PBS (pH 7.4) and shaken for 5 min. Subsequently, the slides were incubated with 3% H_2_O_2_ in the dark for 25 min and washed three times with PBS (pH 7.4) for 5 min each. After pre-incubation with 3% blocking serum for 30 min at room temperature (RT), sections were incubated for 30 min at RT with monoclonal antibodies against rabbit CYP19A1 (1:100) and IR (1:100).The secondary antibody was an HRP-conjugated anti-mouse antibody (SeraCare, Beijing, China), applied for 30 min at a dilution of 1:500. The slides were washed three times with PBS for 5 minutes at RT and slightly dried. The sections were visualized with a chromogenic agent for 5 minutes and mounted.

### Assessment of serum insulin levels

Serum was collected and stored at −70℃ before analysis. Insulin levels were determined by ultrasensitive rat-specific ELISA (Enzyme-free Biotechnology, China) following the instructions present in the ELISA kit. Briefly, all the reagents were left for 30 minutes to equilibrate to room temperature (i.e.,18–25℃). Standard reagents were prepared by serially diluting (1:20) the reagents with distilled water. All the wells of a 96-well plate were filled with 50 μL of the appropriate liquid according to their respective wells (i.e., blank wells, standard wells, and samples). Standard reagents were prepared by serial 5-fold dilution (starting from 1:20) with distilled water. Each well received 50 μL of the diluted standards.. The microplate was firmly sealed and incubated at 37℃ for 30 min. After incubation, the microplate was thoroughly washed 5 times and 50 μL of chromogenic agent A and chromogenic agent B were added. The microplate was gently shaken and mixed well for 10 minutes at 37℃ in a dark room. After 10 minutes, 50 μL of stop reagent was added to terminate the reaction and the absorbance was measured at 450nm using the microplate reader (USCNK, Wuhan, Hubei).

### Assessment of growth plate aromatase activity

Assessments of aromatase in both the obese and control groups were performed as described previously [15]. Briefly, sufficient quantity of growth plate tissue was weighed, mixed with 3x L-1 PBS (pH 7.4, 0.1 M.), homogenized and centrifuged at 10000 × g for 15 minutes at 4 °C. Further, 250 μL of the supernatant was collected.Testosterone (2 μg/mL) and NADPH (3 mg/mL) were added, and the mixture was incubated at 37 °C for 30 minutes. The reaction was terminated in a cold bath. The mixture was then centrifuged at 10000 × g for 30 minutes, and estradiol in the supernatant was estimated by radioimmunoassay and protein content was determined by Bradford assay. The enzyme activity is expressed as the amount of estradiol produced per milligram of tissue protein per hour (pg·mg-¹·h-¹).

### Quantitative real-time PCR

At the end of the culture period, total RNA was extracted from chondrocytes using Quantagene Q225 Real-Time PCR Detection System (Kubo Technology, Beijing, China). The recovered RNA was reverse-transcribed into cDNA. All primers (listed in Table 1) were designed by the NCBI website and synthesized by Wuhan Tianyi Huayu Gene Technology Co., Ltd.1 μg of total RNA and 1 μL of oligo (dT)18 primer were incubated for 5 min at 25℃, followed by incubation for 30 min at 42℃ in the presence of reverse transcriptase. Real-time quantitative PCR was carried out using the same system in a final volume of 25 μL containing 1 μL cDNA, 12.5 μL 2XSYBR Green master mix (Shanghai,Yeasen BioTechnologies) and 0.1μM primers in deoxyribonuclease-free water. The qRT-PCR cycling process was conducted as follows: initial denaturation at 95℃ for 30 s followed by 40 cycles of denaturation at 95℃ for 15 s, annealing at 60℃ for 30 s and further by single cycle of melting curve at 65–105℃.

**Table 1:** Sequence of the primers used in this study.

Primer name	Sequence (5’ to 3’)
R-GAPDH-F	AGACAGCCGCATCTTCTTGT
R-GAPDH-R	CTTGCCGTGGGTAGAGTCAT
R-CYP19A1-F	GACGAGATCGAAATTCTGGTGG
R-CYP19A1-R	CAGGTCTCCACGTCTCTCAG
R-IR-F	CCTTCTCGCGGAGTATGTCC
R-IR-R	ATTCCCGGGCACACCTCTC

CYP19A1, Cytochrome P450 Family 19 Subfamily A Member 1; F, forward; GAPDH,Glyceraldehyde-3-phosphate dehydrogenase; IR, insulin receptors; R, reverse.

### Western Blot analysis

Cultured adherent chondrocytes were washed twice with TBS buffer, lysed with appropriate total cell protein extraction solution, centrifuged at 13,000 g at 4℃ for 5 min, and the supernatant was collected. To evaluate the expression of aromatase and insulin receptors in cultured chondrocytes, whole-cell lysates were solubilized with 1% sodium dodecyl sulfate (SDS) sample buffer and electrophoresed on a 4–15% SDS-PAGE gel. At the end of the in vivo study, proteins were extracted from the tibial growth plates, solubilized with 1% SDS sample buffer, and electrophoresed on a 4–15% SDS-PAGE gel. Proteins were transferred onto a nitrocellulose membrane and probed with monoclonal anti-mouse IR antibody. The blots were developed using a horseradish peroxidase-conjugated polyclonal IgG, and images were captured using the ECL SensiCapture image analysis system on the Peiqing JS-1070P Fluorescent & Chemiluminescence Gel Imaging System (JS-1070P, Shanghai Peiqing Technology Co., Ltd).

### Statistical analysis

The experimental results are presented as mean ± standard error of the mean (mean ± SEM). Statistical analysis was performed using SPSS V16.0 software. Independent samples were compared using Student’s t-test, and comparisons among multiple groups were conducted using one-way ANOVA or two-way ANOVA. A p-value < 0.05 was considered statistically significant. The Shapiro-Wilk test was used to verify the normality assumption of the data, and the Levene test was employed to assess the homogeneity of variances of the data.

## Results

### Alterations in morphological characteristics,growth plate aromatase activity and serum insulin levels

**Fig 1:**
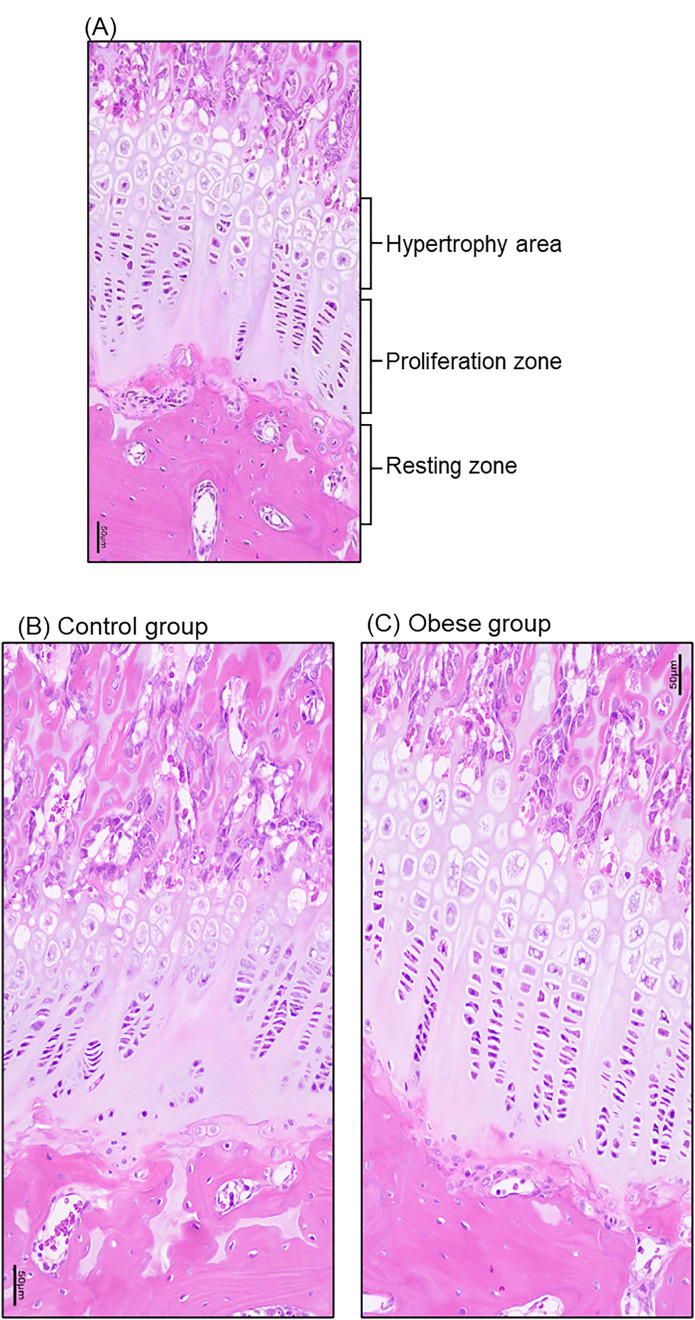
Histology of chondrocytes (A) based on their growth plates and zones (B) control group and (C) obese group. Magnification 200x.

At week 6 of induction, the mean body weight and length were almost similar. By the end of the 6-week induction period, HFD-induced rats showed significant improvement in body weight and length compared to control rats. The results of growth plates and auxological analysis of different areas of growth plates are depicted in Table 2. The height or length of growth plates was significantly greater in obese rats than in control rats. In addition, the proliferative and hypertrophy areas showed significant improvement, while the changes in the rest zone were minimal (Fig 1). Elevated levels of growth plate aromatase activity were prominent in the obese group compared to control group (Table 3.The results of the comparison of serum insulin in obese and control groups are presented in Table 4. Higher levels of serum insulin were observed in the obese group compared to the control group (P < 0.01).

**Table 2:** Measurement of growth plate and analysis of growth zones.

Growth plates and zones	Obese group (μm)	Control group (μm)
Growth plates	426.63 ± 6.28**	331.13 ± 28.93
Rest zone	98.21 ± 9.14	94.43 ± 27.45
Proliferative zone	181.06 ± 23.39*	120.43 ± 19.98
Hypertrophy area	147.36 ± 20.94	116.27 ± 21.11

*p < 0.05, **p < 0.01.

**Table 3:** Comparison of growth plate aromatase activity between obese and control.

Groups	Aromatase activity (U)
Control group	41.1161 ± 1.50235
Obese group	47.2885 ± 0.86523**

*p < 0.05, **p < 0.01.

**Table 4:** Comparison of serum insulin levels between obese and control.

Groups	INS concentration (mU/L)^#^
Control group	22.96 ± 1.98
Obese group	36.46 ± 1.68**

### Immunohistopathological analysis

**Fig 2:**
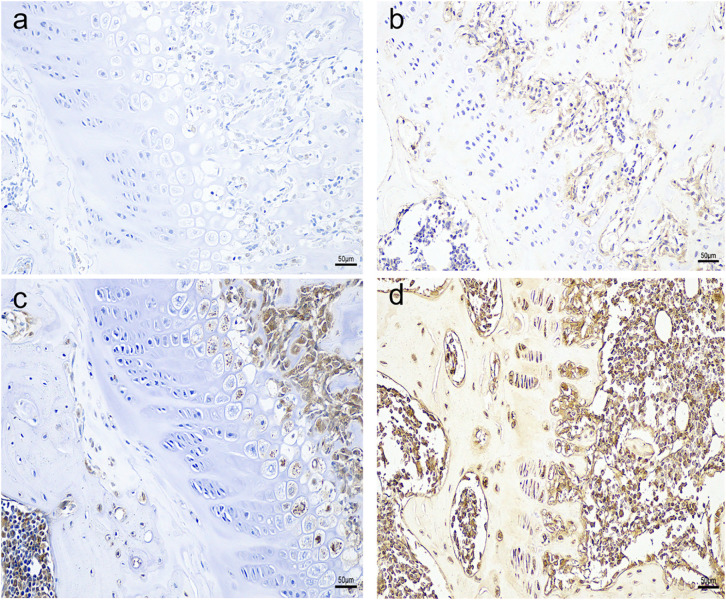
Immunohistochemistry results of aromatase (a.control group,b.obese group) and insulin receptor (c. control group,d.obese group) in SD rats. Magnification of 200x (scale – 50μm).

Immunohistochemistry results revealed that compared with the control group, the growth plates of obese rats showed higher expression of aromatase and strong positive insulin receptor expression in hypertrophic regions (Figure 2).This was further confirmed by a highly significant difference in average OD values in the growth plates of the obese group compared to the control group.

### qPCR and western blot analysis of growth plates for detecting the expression of CYP19A1 and insulin receptors

**Fig 3:**
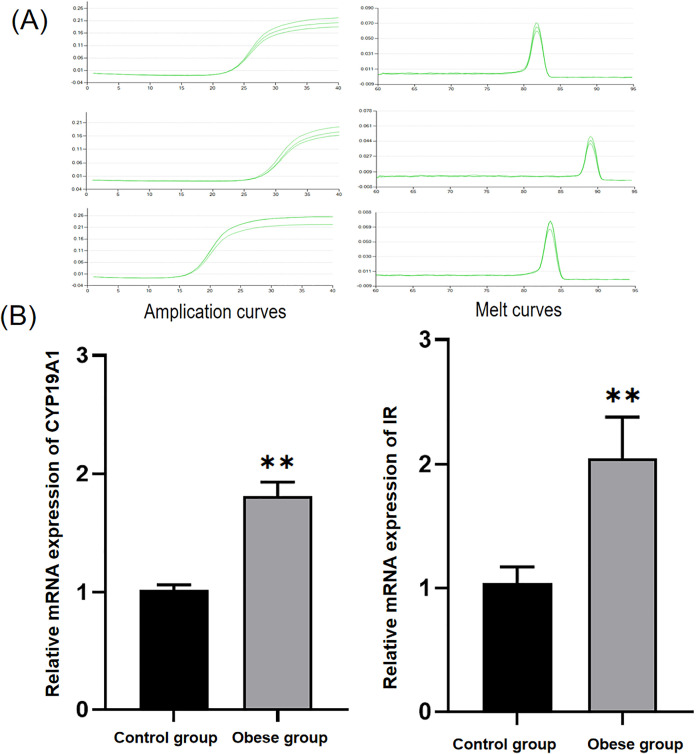
(A) Amplication and melt curves (B) Relative mRNA expression of IR and CYP19A1.

**Fig 4:**
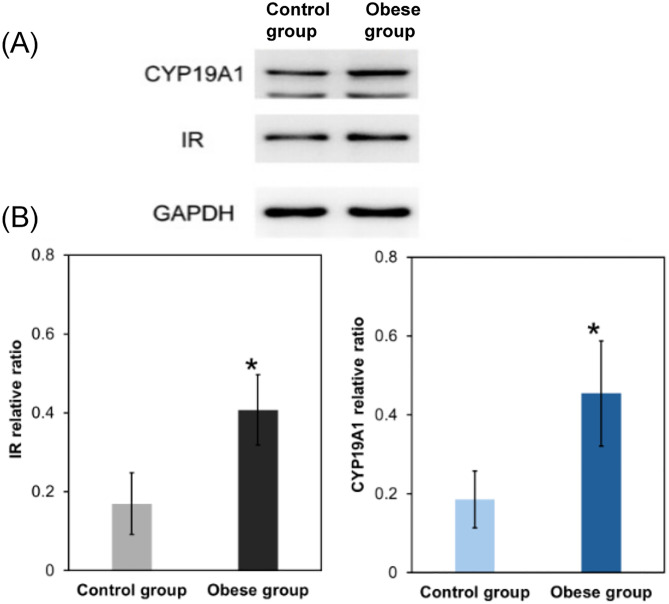
(A) electrophoresis results (B) quantification of IR and CYP19A1.

The total RNA purity at OD260/280 was 1.88–1.97 and OD260/230 was 1.94–2, indicating that purity was in the standard range. The solubility curves of endogenous reference and target genes showed a good unimodal peak with good specificity.The relative mRNA expression of CYP19A1 and insulin receptors (IR) in the growth plates of obese rats was significantly higher than that in the control group (Table 5 and Fig 3). All data from the western blot were presented and analyzed after chemiphotometer exposure. The results showed that significantly higher expression of aromatase and insulin receptors was observed in the growth plates of the obese group compared with the control group (Table 6 and Fig 4).

**Table 5:** The results of qPCR for CYP19A1 and IR in two groups.

	Obese group	Control group
CYP19A1	1.8062 ± 0.12176**	1.0181 ± 0.0436
IR	2.0466 ± 0.33507**	1.0387 ± 0.1314

**Table 6:** The results of Western blot analysis for CYP19A1 and IR in t two groups.

	Obese group	Control group
CYP19A1	0.4544 ± 0.13336*	0.1857 ± 0.07214
IR	0.4069 ± 0.08919*	0.1697 ± 0.07768

## Discussion

Clinical observations have revealed that obese children often exhibit advanced bone age, which consequently impairs their adult height potential. To investigate the underlying mechanism, this study established an obese animal model. While analyzing serum insulin levels, we focused on examining the effects of obesity on the expression of aromatase and insulin receptors in the epiphyseal growth plate.This study established a high-fat diet (HFD)-induced obese rat model to investigate the mechanistic links between obesity-related hyperinsulinemia, growth plate development, and local aromatase expression. The key findings—elevated serum insulin, increased growth plate length (especially in proliferative and hypertrophic zones), and upregulated insulin receptor (IR) and aromatase (CYP19A1) expression in the growth plate—provide critical insights into how obesity may accelerate bone maturation and potentially compromise adult height, while also highlighting unresolved questions for future research.

Consistent with clinical observations in obese children, HFD-fed rats exhibited accelerated linear growth (reflected by greater body length and tibial/humeral bone length) and advanced growth plate maturation at 6 weeks post-induction. Notably, the growth plate in obese rats was significantly longer (426.63 ± 6.28 μm vs. 331.13 ± 28.93 μm in controls, p < 0.01), with the most prominent changes in the proliferative (181.06 ± 23.39 μm vs. 120.43 ± 19.98 μm, p < 0.01) and hypertrophic zones (147.36 ± 20.94 μm vs. 116.27 ± 21.11 μm). This regional specificity aligns with prior studies indicating that the proliferative zone drives longitudinal bone growth via chondrocyte cloning, while the hypertrophic zone mediates terminal differentiation and matrix mineralization—two processes central to growth plate senescence and fusion [16–18]. The minimal change in the resting zone (98.21 ± 9.14 μm vs. 94.43 ± 27.45 μm) is particularly intriguing: as the “progenitor reserve” of the growth plate, the resting zone’s stability suggests that obesity may initially amplify the activity of existing proliferative/hypertrophic chondrocytes rather than depleting stem-like cells—a pattern distinct from estrogen-induced irreversible resting zone exhaustion reported in rabbits [19,20]. This difference may reflect the early stage of obesity in our model (6 weeks) or species-specific responses to metabolic stress.A critical novel observation was the coordinated upregulation of serum insulin, growth plate IR, and aromatase in obese rats. Serum insulin levels were 58.8% higher in the obese group (36.46 ± 1.69 mU/L vs. 22.97 ± 1.99 mU/L, p < 0.01), while IR expression (at both mRNA and protein levels) was significantly elevated in the growth plate—with immunohistochemical staining localizing IR to the hypertrophic zone. Concurrently, aromatase activity (47.29 ± 0.87 U vs. 41.12 ± 1.50 U, p < 0.01) and CYP19A1 expression were markedly increased, and aromatase was also concentrated in the hypertrophic zone. These data support a working model where obesity-induced hyperinsulinemia activates IR-mediated signaling in growth plate chondrocytes, which in turn upregulates aromatase—ultimately accelerating estrogen synthesis locally. This aligns with prior reports that insulin can stimulate aromatase activity in human endometrial stroma [12] and that aromatase expression in rat growth plates increases with sexual maturation [21], but extends these findings to link metabolic (insulin) and endocrine (estrogen) pathways in obesity-related bone maturation.

The colocalization of IR and aromatase in the hypertrophic zone further suggests a cell-specific regulatory axis. Hypertrophic chondrocytes are not only responsible for matrix calcification but also secrete factors like vascular endothelial growth factor (VEGF) that mediate vascular invasion and bone replacement [22,23]. By upregulating aromatase in these cells, insulin may enhance local estrogen production to promote terminal chondrocyte differentiation and matrix mineralization—two steps that precede growth plate fusion. This is consistent with clinical studies showing that obese children with hyperinsulinemia have advanced bone age [24,25] and that estrogen is the primary driver of growth plate closure in both sexes [26]. However, our study cannot definitively confirm that insulin-induced aromatase upregulation is the direct cause of accelerated growth plate maturation: future studies using aromatase inhibitors (e.g., letrozole) or IR antagonists in obese rats would be needed to establish causality.

HFD-induced obese rats exhibited persistent hyperinsulinemia, and insulin resistance along with increased aromatase levels is associated with accelerated growth plates growth. We observed a significant increase in serum insulin levels and insulin receptor expression, indicating that insulin signaling regulates aromatase expression, thereby affecting growth plate development. However, the molecular mechanisms by which insulin receptors modulate aromatase expression remain unclear.This study only investigated growth plate changes in the early stage of obesity (6 weeks) and did not track growth plate closure after puberty. Additionally, the upstream-downstream relationship between insulin and aromatase has not been verified through knockout or inhibitor experiments. Future studies should combine cell models (e.g., primary chondrocyte cultures) and gene editing techniques to clarify the specific regulatory network of the insulin-IR-aromatase pathway in growth plate homeostasis.

## Conclusion

This study demonstrates that obesity-induced hyperinsulinemia accelerates epiphyseal growth plate maturation through aromatase upregulation, highlighting the insulin-aromatase axis as a key regulator of skeletal growth. While further research is needed to elucidate how insulin receptors control aromatase expression, these findings identify novel therapeutic targets for obesity-related growth disorders.

## Supporting information

S1 File The original images and manipulated images of this article. (PDF)
