# Supplementary material for: Investigating insulin’s role in regulating local aromatase in growth plate development
Source: PLoS One. 2025 Dec 2;20(12):e0337215. doi: 10.1371/journal.pone.0337215 (PMC12671795; doi:10.1371/journal.pone.0337215)
Supplement: S1 File — (PDF) [file pone.0337215.s001.pdf]

Supporting Information

Original image　　Original image　　Manipulated image

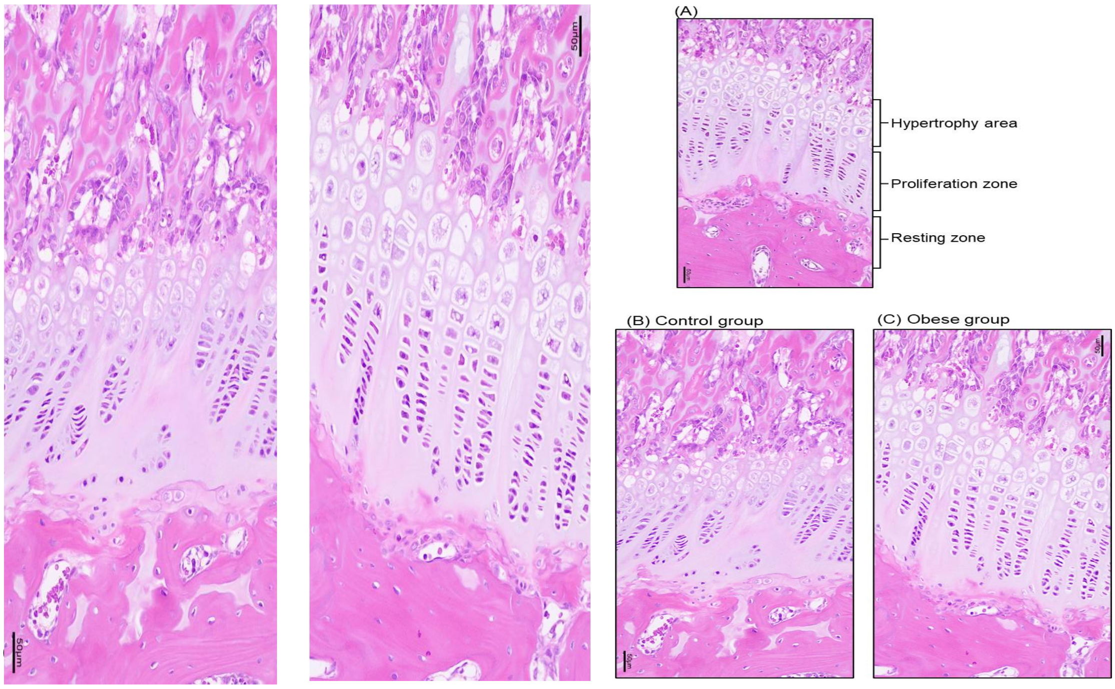

（C）Control group　　（C）obese group

S1-raw-imagines. Histology of chondrocytes **(A)** based on their growth plates and zones **(B)** control group and **(C)** obese group. Magnification 200x.

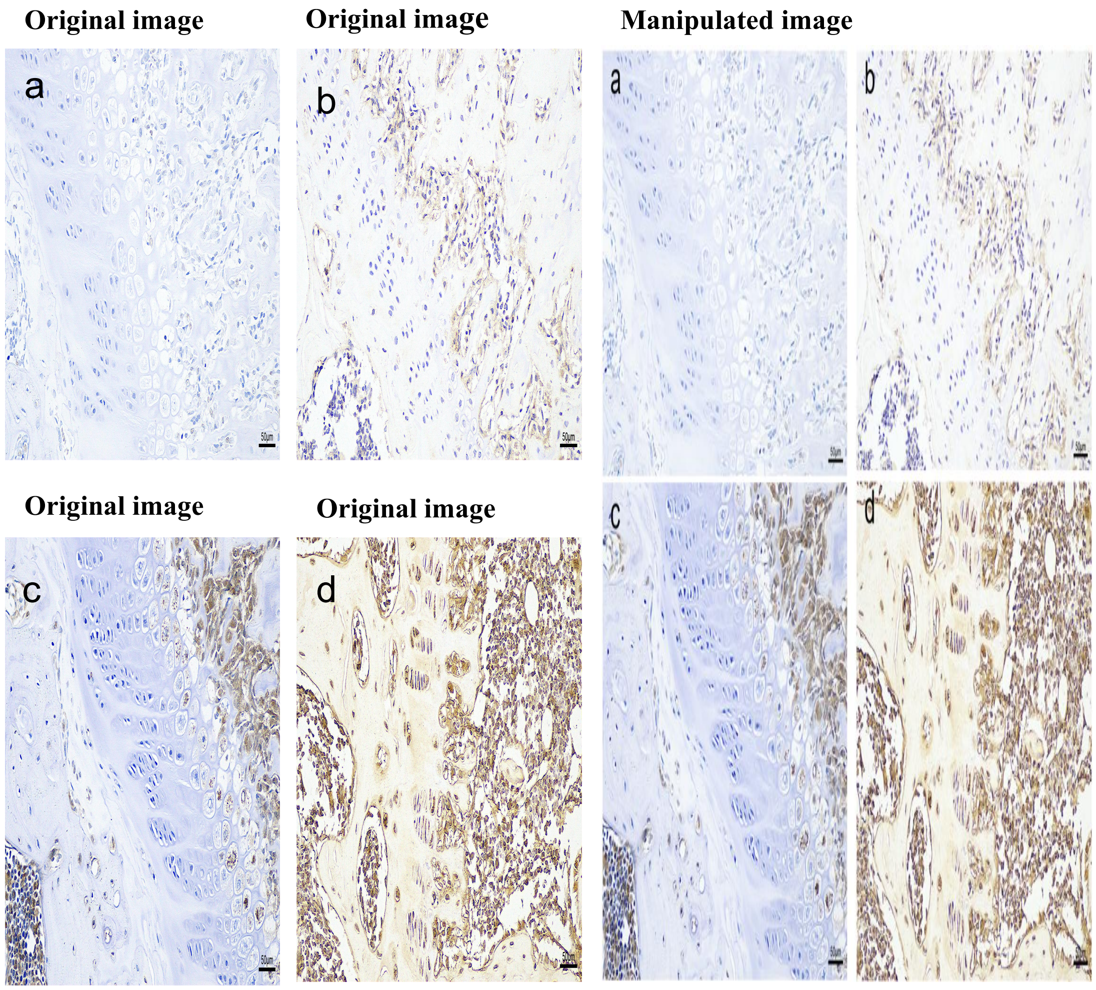

S2-raw-imagines. Immunohistochemistry results of aromatase(a.control group,b.obese group） and insulin receptor（c.control group,d.obese group） in SD rats. Magnification of 200x (scale - 50µm).

CYP19A1 Original image

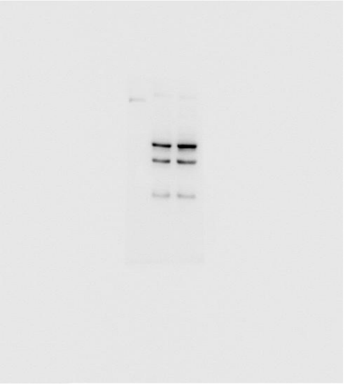

IR Original image

GAPDH Original image

Manipulated image

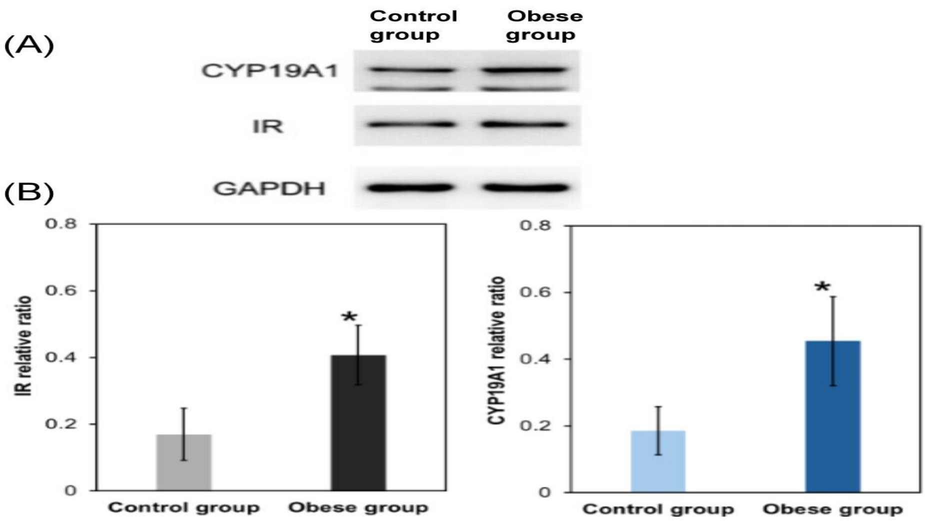

S3-raw-imagines. (A) electrophoresis results (B) quantification of IR and CYP19A1.
